# Dual-degree pathways in the residency match: a comparative analysis of application behaviors and outcomes

**DOI:** 10.1172/jci.insight.198778

**Published:** 2026-02-19

**Authors:** Daniel C. Brock, Deborah D. Rupert, Toni Darville, Caroline S. Jansen, Elias M. Wisdom, Cynthia Y. Tang

**Affiliations:** 1 American Physician Scientists Association, Boxborough, Massachusetts, USA.; 2 Medical Scientist Training Program, Baylor College of Medicine, Houston, Texas, USA.; 3 Department of Anesthesiology, Washington University School of Medicine in St. Louis, St. Louis, Missouri, USA.; 4 Department of Pediatrics and; 5 Medical Scientist Training Program, University of North Carolina at Chapel Hill, Chapel Hill, North Carolina, USA.; 6 Department of Internal Medicine, Yale School of Medicine, New Haven, Connecticut, USA.; 7 Medical Scientist Training Program, Oregon Health & Science University, Portland, Oregon, USA.

## Abstract

Dual-degree medical students pursue additional training to prepare for careers in research, public health, and administration, but how these experiences influence residency application behaviors and outcomes are poorly understood. We analyzed 36,298 residency applicants from the Texas Seeking Transparency in Application to Residency (TexasSTAR) database spanning 2017–2023 to compare application, interview, and match patterns among single-degree MD applicants and those with MD-PhD, MD-MPH, MD-MBA, or MD-MSc degrees. Despite differences in academic metrics, application strategies, and interview rates, match rates were similar across degree groups. MD-PhD students applied to fewer programs but had the highest interview offer–to–application rate and matched at more prestigious programs based on Doximity rankings. Beyond traditional application metrics such as board scores, research productivity, grades, and honor society membership, strategies including away rotations, geographic preferencing, and program signaling were associated with increased interview offers and match success among all applicants but were less influential for dual-degree applicants. These findings suggest dual-degree applicants require specialized advising and evaluation.

## Abstract 2

Dual-degree training shapes residency application strategies, interview rates, specialty choice, and program prestige. Read the full analysis here.

## Introduction

The residency match is a pivotal step that shapes the immediate postgraduate experiences and long-term career trajectories of medical students. Residency programs evaluate applicants using a range of factors, including academic performance, board scores, research productivity, leadership roles, and extracurricular activities (1). However, the process has become increasingly competitive, imposing financial, emotional, and logistical burdens on both applicants and programs (2). While existing studies have focused on associations between applicant metrics and match outcomes among traditional doctor of medicine (MD) and doctor of osteopathic medicine (DO) candidates (3–7), there are limited data examining the distinct challenges and outcomes for dual-degree applicants (8), particularly with recent changes to the residency application.

Dual-degree physicians serve a critical role in leading and bridging clinical care with broader healthcare needs (9, 10), with expertise spanning biomedical research, public health, healthcare management, and data science. To obtain additional skills or gain a competitive edge, some medical students pursue dual-degree programs, such as a doctor of philosophy (PhD, 2%–3%) (11), master of public health (MPH, 3%) (12), master of business administration (MBA, 1%) (13), or master of science (MSc, 1%–2%) (14). The percentage of medical schools offering dual-degree options has steadily increased over the past 20 years, reflecting growing interest in these specialized training opportunities (12, 14–16). Despite these trends, the effect of dual-degree status on residency application strategies and match outcomes remain understudied. Existing national datasets from the Association of American Medical Colleges (AAMC) or National Resident Matching Program (NRMP) do not consistently disaggregate outcomes by degree type in publicly available data. Dual-degree trainees may face unique challenges, including extended training timelines and potential negative perceptions from residency programs (17). Consequently, there is a lack of clarity regarding how specific groups of dual-degree students should approach the residency application process.

To address these knowledge gaps, we analyzed 7 years of national, self-reported data from the Texas Seeking Transparency in Application to Residency (TexasSTAR) survey (18). We compared dual-degree residency applicants with their MD-only peers from allopathic medical schools in the United States. Specifically, we analyzed: (a) applicant demographic profiles, academic performance, and extracurricular activities of each applicant group; (b) success in securing residency interview offers and match placements; and (c) the influence of the evolving application process with program signaling and geographic connections. This study provides a comprehensive update to prior publications by exploring multiple dual-degree types across several application cycles, with new analyses on interview offer rates, match rates, away rotations, program signaling, and geographic connections (10, 19–22).

Our findings can inform dual-degree program selection for prospective medical students, support current dual-degree students in preparing their residency applications, facilitate tailored advising during the residency application process, and provide insights to residency programs into the interests of dual-degree applicants, helping to improve evaluation and recruitment for this uniquely trained cohort. Together, this study underscores the importance of developing more individualized approaches to residency in an evolving medical education landscape.

## Results

### TexasSTAR respondent demographics.

This study analyzed responses from 36,298 fourth-year medical students at US-accredited MD programs, including 30,179 MD; 1,239 MD-PhD; 1,797 MD-MPH; 532 MD-MBA; and 2,551 MD-MSc students. We compared demographics of dual-degree students to their MD-only counterparts (Table 1). MD-PhD students were significantly older: 97.2% were over 28 years old (yo), compared with 24.3% of MD-only students. Similar but less pronounced trends were observed among MD-MPH, MD-MBA, and MD-MSc students. The MD-PhD cohort had a 13.2% lower proportion of female students compared with MD-only students, while the MD-MBA group had a 23% lower proportion. In contrast, the MD-MPH cohort had 9% more female students.

Compared with MD-only students, MD-MPH students were more likely to identify as underrepresented in medicine (UriM) (odds ratio [OR] = 1.61 [95% CI, 1.29–2.00]), as were MD-MSc students (OR = 1.43 [95% CI, 1.17–1.74]) (Table 1). First-generation college student status did not differ significantly across groups.

### Application metrics.

Application metrics include United States Medical Licensing Examination (USMLE) scores, academic outputs (research experiences, presentations, and peer-reviewed publications), extracurricular activities (volunteer and leadership activities), grades, and honor societies (Alpha Omega Alpha [AOA] and Gold Humanism Honors Society [GHHS]). Performance on the USMLE Step 1 and Step 2 serves as a critical determinant of residency match success (23–25). We analyzed numeric Step 1 scores prior to the exam’s transition to pass/fail in January 2022. MD-PhD students had a 2-points higher mean Step 1 score than MD-only students (Table 1). In contrast, MD-MPH and MD-MSc students had lower mean Step 1 scores. For Step 2, all dual-degree groups except MD-MPH students had lower mean scores than MD-only students (1.9–3.9 points lower).

All dual-degree groups reported significantly more research experiences, presentations, and peer-reviewed publications, compared with MD-only students (Table 1). MD-PhD students reported fewer volunteer activities, while MD-MPH students reported more. MD-MPH, MD-MBA, and MD-MSc students reported a significantly higher number of leadership roles. All dual-degree groups were less likely than MD-only students to achieve honors in clinical clerkships. However, MD-PhD students displayed a 5.2% higher rate of achieving honors in their chosen specialty. At institutions with AOA chapters, MD-MPH and MD-MSc students had lower induction rates than MD-only students by 4.3% and 4.0%, respectively. For institutions with GHHS chapters, MD-PhD students showed lower induction rates (9.3% versus 17%; OR = 0.50 [0.41–0.61]), while MD-MPH students showed higher rates (25.7% versus 17%; OR = 1.68 [1.5–1.88]), consistent with prior reports (26). Overall, dual-degree applicants displayed distinct application metrics compared with their single-degree counterparts.

### Application volume, interview rates, and match outcomes.

**Figure 1:**
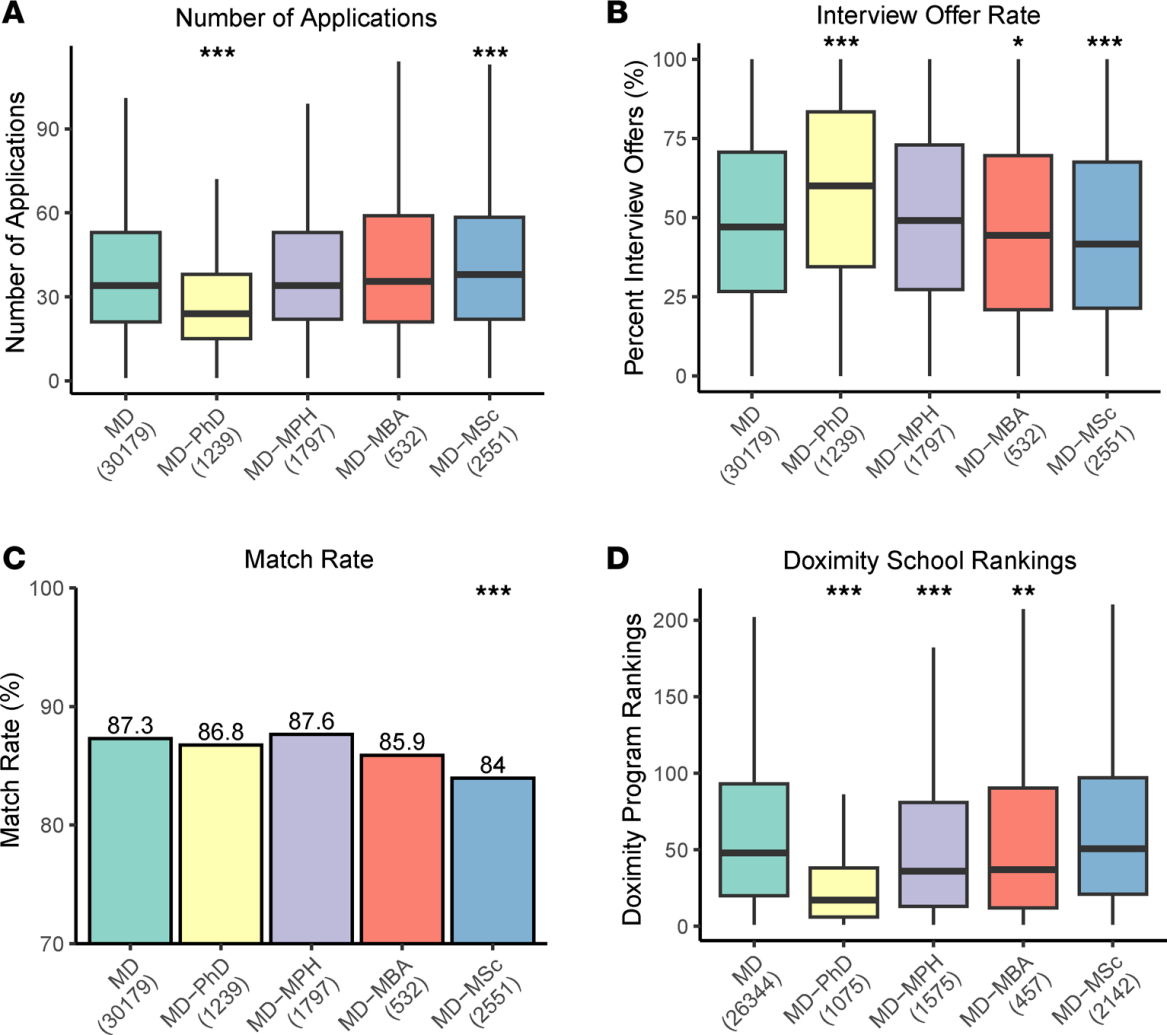
Comparison of application volume, interview offers, and match rates of dual-degree applicants to MD-only applicants. (**A**–**D**) Number of applications submitted, rate of interview offer–to–applications submitted (%), match rate (%), and median Doximity rankings of matched programs. In **D**, lower values reflect higher-ranked programs. All applicants, including those listing transitional and preliminary years as their specialties, were included in analyses. Figures are shown as box-and-whisker plots. The horizontal line within each box indicates median, box edges denote interquartile range (IQR), and whiskers extend to 1.5 × IQR. Statistical significance was analyzed using Wilcoxon signed-rank tests with FDR correction, comparing each dual-degree group with the MD-only group. Match rate comparisons were evaluated using Fisher’s exact test. *P < 0.05, **P < 0.001, ***P < 0.0001. Values below the horizontal axis labels indicate number of students per group.

Next, we examined application, interview, and match patterns across degree pathways. MD-only students applied to a median of 34 programs (interquartile range [IQR], 21–53) (Figure 1A). In comparison, MD-PhD students applied to 29% fewer programs, with a median of 24 applications (IQR, 15–38). MD-MSc students applied to a median of 38 (IQR, 22–59).

To account for differences in application volume, we calculated the interview offer rate as the percentage of interview offers received relative to the number of applications submitted (Figure 1B). MD-only students had a median interview offer rate of 47.1% (IQR, 26.7–70.6). MD-PhD students had a significantly higher offer rate of 60% (IQR, 34.5–83.3). In contrast, MD-MBA and MD-MSc students reported slightly lower interview offer rates of 44.4% (IQR, 20.9–69.6) and 41.7% (IQR, 21.4–67.6), respectively. Despite differences in interview offer rates, overall residency match rates were similar across degree pathways, ranging from 84% to 88% (Figure 1C). MD-MSc students had a significantly lower match rate at 84%, compared with 87.3% for MD-only students. MD-PhD, MD-MPH, and MD-MBA students did not differ from MD students in overall match success.

To determine whether these patterns changed after the COVID-19 pandemic, we analyzed application volume, interview offer rates, and match rates before the pandemic (2017–2019) and after transition to virtual interviews (2020–2023) (Supplemental Figure 1; supplemental material available online with this article; https://doi.org/10.1172/jci.insight.198778DS1). MD (median 32–35 applicants) and MD-MBA (28.5–36) applicants applied to more programs. Interview offer rates significantly dropped for MD (from 51.4% to 45%), MD-MPH (from 54.5% to 46.7%), and MD-MBA (from 45.6% to 42.9%) applicants. Despite the decreased interview rates, match rates remained unchanged for all degree groups.

We also analyzed institutional rankings from Doximity (27) to determine whether degree pathway was associated with the prestige of matched residency programs, where lower numerical values indicate higher-ranked programs (e.g., rank 1 is the highest). MD-PhD students matched into more prestigious programs, with a median rank of 17 (IQR, 6–38), compared with a median of 48 for MD-only students (IQR, 20–93) (Figure 1D). Similarly, MD-MPH and MD-MBA students matched into significantly higher-ranked programs with median ranks of 36 (IQR, 13–80.8) and 37 (IQR, 12–90.2), respectively. We observed similar trends when using department-specific rankings from the Blue Ridge Institute for Medical Research (6. https://brimr.org/; Horse Shoe, North Carolina, USA) (Supplemental Figure 2).

### Away rotations, geographic connections, and signaling.

**Figure 2:**
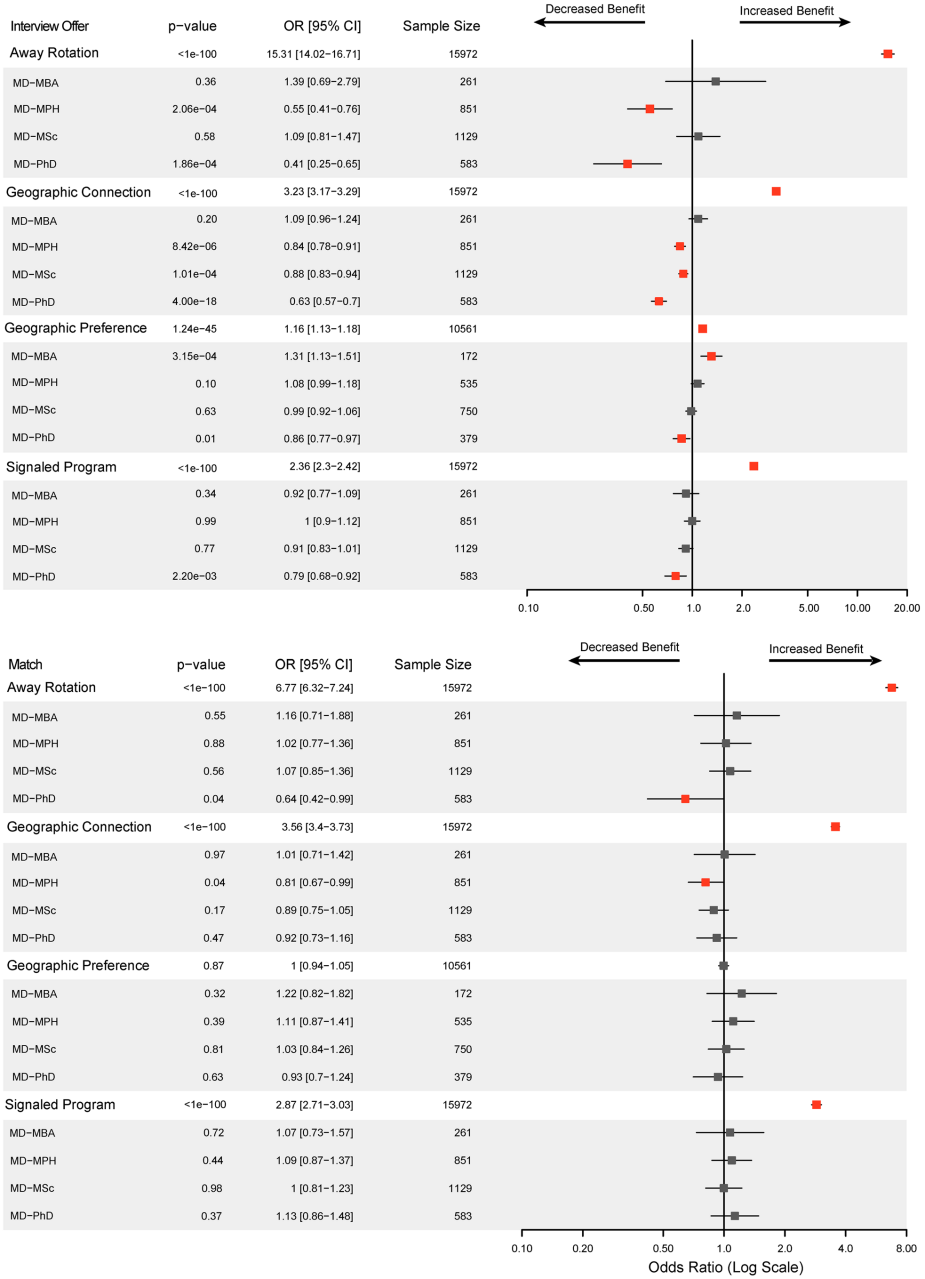
Modifiable residency application strategies. Multivariable logistic regression was used to assess the impact of away rotations, geographic connections, geographic preferencing, and program signaling on interview offers and match success for each degree path compared with MD-only. The overall cohort was used to generate the main effects (white rows). Dual-degree status was also included as an interaction term compared with MD-only (gray rows) to evaluate whether incremental effects of these strategies differed in predictive importance by degree path, relative to MD-only applicants. For interaction terms, an odds ratios (OR) < 1 indicates reduced marginal benefit compared with MD-only applicants, whereas OR > 1 indicates increased benefit. Squares represent OR, lines represent 95% CI, and red indicates statistical significance (*P* < 0.05).

With the recent implementation of geographic connections, geographic preferencing, and program signaling, as well as increasing interest in away rotations, we examined how applicants influenced their interview and match outcomes through modifiable application strategies, including away rotations, geographic connections, geographic preferencing, and program signaling (Figure 2). When applicants were analyzed together in a multivariable logistic regression model, completing an away rotation increased the odds of receiving an interview offer by 15.3-fold (95% CI, 14.0–16.7) and the odds of matching by 6.8-fold (95% CI, 6.3–7.2). Applicants with geographic connections, defined by TexasSTAR as a personal link to the region, had an overall 3.23 times (95% CI, 3.17–3.29) higher odds of receiving an interview and 3.56 times (95% CI, 3.40–3.73) higher odds of matching. Geographic connections differ from geographic preference signaling, which was piloted by the AAMC in 2022 and expanded in 2023 (28). Geographic preference signaling had a 1.16-fold (95% CI, 1.13–1.18) increased overall association with receiving an interview invite and no association with matching. Program signaling increased the overall odds of receiving an interview by 2.36-fold (95% CI, 2.30–2.42) and of matching by 2.87-fold (95% CI, 2.71–3.03).

However, when using dual-degree status as an interaction term compared with MD-only, we found that these application strategies had less effect for dual-degree students (Figure 2). Compared with MD-only students, away rotations had a smaller effect on interview offers for MD-PhD (OR = 0.41 [0.25–0.65]) and MD-MPH students (OR = 0.55 [0.41–0.76]), as well as match success for MD-PhD students (OR = 0.64 [0.42–0.99]). Personal geographic connections had less effect for receiving an interview offer for MD-PhD (OR = 0.63 [0.57–0.70]), MD-MPH (OR = 0.84 [0.78–0.91]), and MD-MSc students (OR = 0.88 [0.83–0.94]). For match, only MD-MPH students showed lower benefit from geographic ties (OR = 0.81 [0.67–0.98]). Geographic preference signaling had less benefit for receiving interview invites for MD-PhD students (OR = 0.86 [0.77–0.97]). Only MD-PhD students showed less benefit of program signaling on interview offers (OR = 0.79 [0.68–0.92]). Overall, while program signaling, geographic connections, geographic preferencing, and away rotations were important for overall interview and match success, these components were considerably more influential for MD-only applicants.

### Interview and match patterns across specialties.

**Figure 3:**
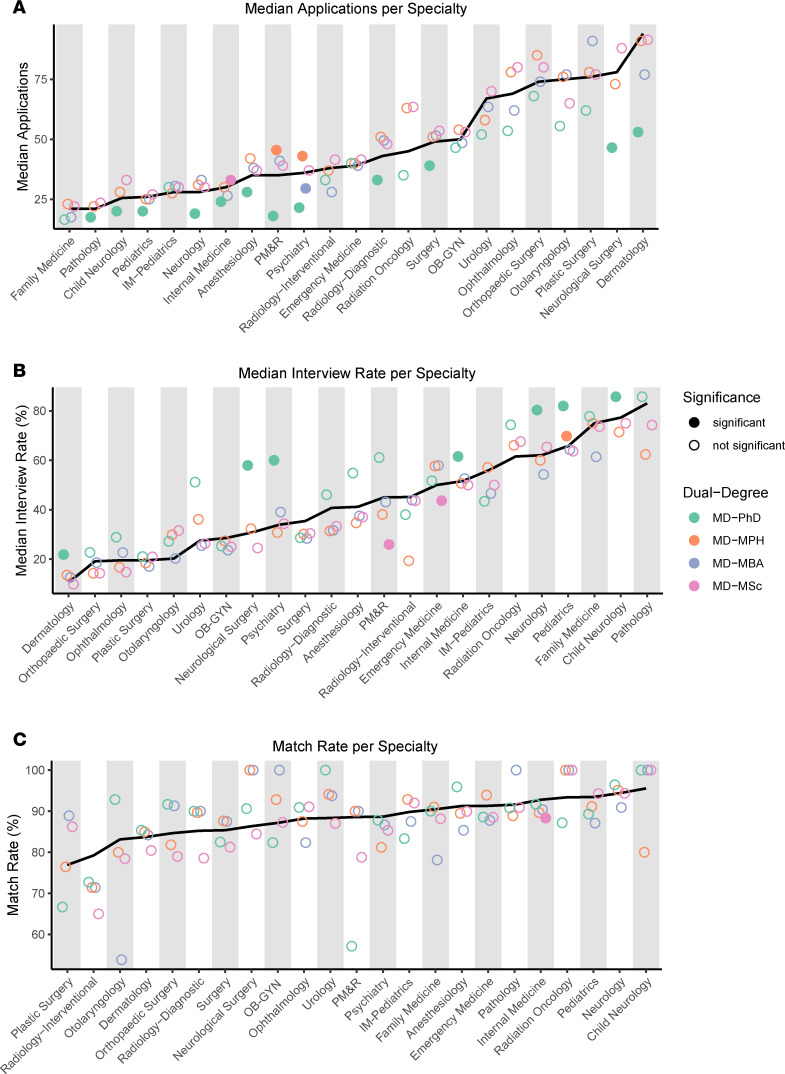
Specialty-level trends in applications, interviews, and match by degree path. (**A**) Median applications submitted per specialty, stratified by dual-degree status. (**B**) Median interview offer rate per specialty. (**C**) Match rate per specialty. The black line represents the MD-only median for each specialty and serves as the reference for comparisons to dual-degree groups. Specialties are sorted from least to greatest MD-only medians for each panel. Statistical significance (*P* < 0.05) for total number of applications and interview offer rates was assessed using Wilcoxon tests, whereas match rates were evaluated using multiple Fisher exact tests with FDR correction. Applicants who listed “Preliminary” or “Transitional” as their specialty were excluded.

Application volume and interview offer rates varied widely by specialty (Supplemental Table 1). We stratified application metrics by degree path and compared each group to MD-only students. MD-PhD students submitted significantly fewer applications across 12 of 23 specialties, reflecting the broader trend of reduced application volume (Figure 3A). These differences were most pronounced in highly competitive specialties, including dermatology, where MD-PhD applicants applied to a median of 41 fewer programs, and in surgical fields, including neurological surgery (31.5 fewer programs) and otolaryngology (19.5 fewer programs). MD-MPH students applied to more programs in physical medicine and rehabilitation (PM&R) and psychiatry.

Dual-degree students also demonstrated varying interview offer rates (Figure 3B). Compared with MD-only students, MD-PhD students demonstrated higher interview offer rates in dermatology, neurosurgery, psychiatry, internal medicine, neurology, pediatrics, and child neurology. MD-MSc students had lower offer rates in PM&R and emergency medicine, while MD-MPH students had higher interview rates in pediatrics. MD-MBA students did not differ in any specialty-specific metric.

Specialty-specific match rates did not differ across most dual-degree groups (Figure 3C). MD-MSc students demonstrated a modestly lower match rate in internal medicine. All other comparisons were not statistically significant. Overall, while application volume and interview offer rates differed across groups, these differences did not translate into differences in match rates.

### Dual-degree students differ by specialty preferences.

To determine whether dual-degree applicants differed in specialty preferences, we compared the specialty distributions of dual-degree applicants with those of MD-only applicants. First, we assessed whether self-reported data from TexasSTAR accurately reflected national patterns in specialty selection. We compared data from TexasSTAR and the AAMC (29), which reports total resident enrollment and MD-PhD enrollment by specialty. AAMC data are only disaggregated for MD-PhD students, permitting its use as a national benchmark. We found that specialty-specific ORs derived from TexasSTAR were highly correlated with those from the AAMC (R^2^ = 0.918; *P* < 0.001) (Supplemental Figure 3 and Supplemental Table 2). These findings show that, despite its self-reported nature, TexasSTAR captures specialty selection trends consistent with national data.

We found that each degree path pursued different medical specialties (Table 2). Compared with MD-only applicants, MD-PhD applicants were more likely to pursue internal medicine, neurosurgery, radiation oncology, dermatology, pathology, neurology, and child neurology but less likely to pursue family medicine, internal medicine-pediatrics, obstetrics and gynecology (OB-GYN), orthopedic surgery, general surgery, anesthesiology, emergency medicine, and PM&R. MD-MPH applicants were more likely to pursue family medicine, internal medicine-pediatrics, OB-GYN, and diagnostic radiology but less likely to pursue ophthalmology, orthopedic surgery, anesthesiology, and dermatology. MD-MSc applicants were more likely to pursue neurosurgery, general surgery, and pathology. MD-MBA and MD-MSc applicants were less likely to pursue pediatrics.

## Discussion

This study provides an in-depth analysis of how dual-degree status influences residency application behaviors, interview rates, and match outcomes across a large cohort of U.S. MD students. Dual-degree trainees face added challenges compared with MD-only counterparts, and recent funding and structural barriers further complicate these training pathways (30). This study analyzed how dual-degree status influenced residency application behaviors, interview rates, and match outcomes across a large cohort of U.S. MD students. Building on previous work examining residency outcomes for dual-degree students (31), this study comprehensively updates these findings in the context of evolving Electronic Residency Application Service (ERAS) components, analyzing over 36,000 applicants. Dual-degree applicants exhibited distinct patterns in demographics, academic metrics, application volume, interview offer rates, and utility of away rotations, geographic connections, and program signaling. These findings support the need for specialized residency advising.

Our study cohort revealed demographic disparities among dual-degree residency applicants. MD-PhD students were older and more likely to identify as male than MD-only peers. This age difference is expected, as MD-PhD training typically requires an additional 4.25 years for PhD completion and many MD-PhD students have also taken research gap years (32). The sex imbalance among MD-PhD applicants aligns with prior research indicating that MD-PhD programs have struggled to recruit women and URiM individuals, though recent data suggest gradual improvement (33–36). MD-MBA applicants also demonstrated a substantial sex disparity, with women comprising only one-third of applicants, consistent with a previous study (37). In contrast, MD-MPH applicants were more likely to identify as female and as URiM, likely reflecting alignment with interest in improving health equity in underserved populations (12, 38). MD-MSc students were older and more likely to be male, yet they were more likely to identify as URiM. This may reflect different paths to obtaining an MSc, which are often earned independently before matriculating to medical school. Applicants may pursue master’s degrees during gap years to strengthen their medical school applications. Overall, these patterns underscore ongoing diversity gaps in dual-degree pathways (39, 40).

Dual-degree students demonstrated higher levels of academic productivity but generally had lower USMLE exam scores compared with MD-only peers. All dual-degree groups had lower Step 2 scores compared with MD-only. When pursued during medical school, MD-MPH, MD-MBA, and MD-MSc pathways typically introduce a 1- to 2-year academic gap, while MD-PhD students often experience a 3- to 6-year gap between Step 1 and Step 2 (41, 42). Clinical refresher bootcamps or longitudinal clinical curricula integrated during PhD training may help mitigate this trend. During clinical training, dual-degree students may also experience added strain from ongoing research or other academic obligations. These gaps may lead to challenges in retaining and relearning preclinical materials, which may slightly adversely affect USMLE exam performance. However, although statistically significant, score differences were small and may lack practical significance.

Residency application approaches also varied by degree pathway. MD-PhD students applied to fewer programs, likely reflecting a more targeted approach focused on research-intensive academic institutions. This strategy is often shaped by a personal interest in an academic career, as well as guidance from peers and mentors that applications to nonresearch-focused programs may yield lower interview rates. Thus, MD-PhD applicants may strategically tailor their applications based on institutional fit and the marketability of their unique training. Consistent with this approach, MD-PhD students had a higher interview offer rate in select specialties and matched into programs with higher Doximity rankings, typically academic medical centers with strong research environments. While the COVID-19 pandemic disrupted traditional residency application patterns as programs shifted to virtual interviews, we did not identify meaningful differences in overall match rates across degree types.

Furthermore, while away rotations, geographic connections, and program signaling are known to influence interview and match outcomes (8, 43), their influence varied by degree pathway. Away rotations were strongly associated with interview and match success for MD-only students but had less effect on dual-degree students. Likely explanations may include specialty-specific requirements and limited scheduling flexibility, particularly for MD-PhD students, whose medical school timelines may be compressed following completion of their PhD. Similarly, geographic connections played a different role for dual-degree applicants. Rather than prioritizing proximity to home, these students may focus on institutional alignment. For example, MD-PhD students may aim for large academic medical centers, and MD-MPH students may target settings where they are better situated to address health disparities. Furthermore, while data have shown clear advantage in interview rates for signaled programs (44), we found that program signaling was very important for MD-only applicants but had less effect for dual-degree applicants. We reason that dual-degree applicants may apply to special residency tracks or programs that value their additional training. Program and geographic signaling, rolled out from 2020 to 2024, are new additions to the ERAS application and as more data become available, additional studies will be necessary to assess its increasing importance.

Finally, as previously reported (10), dual-degree candidates disproportionately pursued certain specialties. MD-PhD students were more likely to pursue neurology, pathology, and radiation oncology, and less likely to apply to surgical or primary care specialties. This underrepresentation raises concerns about the future integration of scientific advancements in these fields, gaps that are unlikely to close unless programs can provide more structured residency pathways offering protected research time. In contrast, MD-MPH students more frequently pursued family medicine, internal medicine-pediatrics, and OB-GYN, which may align with their emphasis on population health and health equity (12). MD-MBA students showed no strong specialty preferences aside from a lower likelihood of pursuing pediatrics.

## Limitations

This study has several limitations. First, while we propose plausible explanations for the observed trends, we cannot directly assess applicant motivations or decision-making processes because survey data were not available. Second, pooling data across 7 application cycles was necessary to ensure adequate statistical power; however, this approach may mask temporal changes related to evolving application practices or policy shifts. Third, our analysis is constrained by the lack of primary data sources. The AAMC does not disaggregate dual-degree applicants. However, we observed strong concordance between AAMC and TexasSTAR data for MD-PhD applicants (Supplemental Figure 3), supporting the reliability of self-reported TexasSTAR outcomes. Participating medical schools had an approximate 40% response rate (18). We observed that unmatched applicants were overrepresented in the TexasSTAR data. According to 2025 NRMP data, 93% of US MD seniors matched, compared with a match rate of 87% in the TexasSTAR data (45). Fourth, Doximity rankings were used as a proxy for program prestige due to its accessibility and wide utility by applicants (46). However, program prestige is subjective. Fifth, while additional degrees are reported in TexasSTAR, it is unclear when the degrees were obtained. While many students choose to pursue an additional degree concurrently with their medical degree, trainees may have also opted to pursue a nonmedical degree prior to starting medical school. Sixth, sample sizes for some dual-degree groups, particularly MD-MBA students, are relatively small. Seventh, TexasSTAR does not offer data on applicant rank lists, which would provide additional context to match success. Finally, while this study offers important population-level insights, findings should not be extrapolated to individual applicants.

## Conclusions

Our findings have important implications for dual-degree medical trainees, residency programs, and institutions. The residency match is complex for dual-degree students, who must balance traditional application demands with the added nuances of their specialized training. This analysis highlights differences in demographics, academic performance, interview rates, and application patterns for dual-degree students. Overall, we identified 4 key findings: (a) despite differences in USMLE scores, academic performance, and research productivity, match rates were comparable across degree types; (b) MD-PhD students applied more selectively but received proportionally more interviews and matched at higher ranked programs; (c) modifiable application strategies, including away rotations, geographic preferencing, and program signaling, benefited all applicants but had a greater effect on MD-only applicants; and (d) degree pathway influenced specialty choice.

Due to these differences, institutions should provide specialized match advisors for dual-degree students, ideally similarly trained physicians. Residency programs interested in recruiting dual-degree applicants should adjust their reviews of these applicants with the understanding that, while they may have fewer clerkship honors and honor society memberships than their MD-only peers, they tend to bring increased academic productivity and leadership in addition to their specialized training. Trainees should seek mentorship from physicians with similar career trajectories both within and beyond their institutions, emphasize their academic fit with the residency program, provide a clear leadership narrative, and submit strong letters of recommendation that support their clinical proficiency. Overall, these insights may inform efforts to better support and retain dual-degree trainees.

## Methods

### Study population.

Individual-level data from 40,039 applicants from 146 US-accredited medical schools was available in the TexasSTAR survey from 2017 to 2023 (18). The TexasSTAR database is a national survey and online tool designed to assist medical students in tailoring their residency applications. It collects self-reported data from fourth-year medical students. Over our 7-year study period, the overall response rate was 40.8%, calculated using annual application statistics from the ERAS (47).

Our 7-year retrospective observational study focused on MD students, excluding those with dual DO degrees (*n* = 1,695) due to the limited sample size of dual-degree DO students. Thus, our analyzed study population consisted of 36,298 MD students categorized into the following groups: MD-only, MD-PhD, MD-MPH, MD-MBA, and MD-MSc. TexasSTAR does not report MD-JD students separately; instead, they are grouped under the broader category of “MD-other,” which includes all additional degree types not explicitly classified. MD-other degree paths were not analyzed in this study.

The primary outcomes for this study were number of applications submitted, interview offer rates, and match rates. Degree path descriptor variables were derived from the ERAS application, including demographics, USMLE Step scores, honors society memberships (AOA, GHHS), academic performance indicators (clerkship honors), research productivity (publications, presentations, abstracts), volunteer experiences, and leadership roles. Predictor variables for interview offers and matching included away rotations, geographic connections, geographic preference signaling, and program signaling. Overall analyses by degree path included all eligible applicants. Specialty-level analyses excluded specialties with fewer than 5 respondents and individuals that listed transitional or preliminary years as their specialty.

### Additional data sources.

Data from the AAMC were used to validate self-reported information in TexasSTAR against ERAS application trends for MD-PhD students (29) (Supplemental Figure 3). Additional data for MD-only and MD-PhD applicants were extracted from the AAMC.

Residency program rankings by reputation and research output were gathered using the Doximity Residency Navigator (27) accessed in December 2024. Not all specialties had ranked programs, and not every program in TexasSTAR was ranked. Overall, 93.6% and 88.8% of programs were successfully matched to Doximity reputation and Doximity research output rankings, respectively. Given the high concordance between the 2 ranking systems, Doximity reputation rankings were used for all analyses in this study. While multiple ranking systems exist, Doximity rankings were used here, as they are a publicly available and widely used metric for residency applicants (46, 48). Specialty-specific federal funding–based rankings from the Blue Ridge Institute for Medical Research were used to corroborate Doximity residency rankings; however, coverage was limited, as only 22% of matched applicants could be mapped to a Blue Ridge–ranked program.

### Statistics.

The Shapiro-Wilk test was used to assess normality of continuous variables, while Levene’s test evaluated homogeneity of variances. Categorical variables including age group, sex, race, ethnicity, first-generation status, couples matching, honors society memberships, honors in specialty of choice, specialty selection, and match status were analyzed using Fisher’s exact test. For categorical variables with more than 2 levels, pairwise Fisher’s exact tests were conducted, with multiple hypothesis correction applied using FDR. Kruskal-Wallis tests were used to compare continuous variables across degree groups (i.e., Step scores, research experiences, research outputs, volunteer experiences, leadership roles, clerkship honors, number of applications, interview offers, and Doximity residency rankings). Post hoc comparisons for these variables were conducted using Wilcoxon signed-rank test with FDR correction. For Step scores, which approximated a normal distribution, 1-way ANOVA was used, followed by Tukey’s test for post hoc pairwise comparisons.

To preserve applicant anonymity, personally identifiable data (i.e., exact age, home medical school, and raw Step scores) were not reported. TexasSTAR records age in ranges; thus, age was analyzed as a categorical variable. TexasSTAR records Step 1 and Step 2 scores in binned increments of 5 (e.g., 250–254). We centered these increments and treated them as continuous interval data. For instance, a Step 1 score in the 250–254 bin was recorded as 252.

Medians and IQR were reported for the number of applications and interview offers, as they were not normally distributed. We calculated the interview offer rate, defined as the percentage of the number of interview offers divided by the total number of applications submitted. This rate was also calculated at the specialty level and used as a normalized outcome measure. In all comparisons, MD-only students served as the reference group compared with dual-degree groups.

To assess the effect of modifier variables on interview offers and match rates, we performed logistic regression for away rotations, geographic connections, geographic preference, and program signaling. We first generated univariable summaries of these variables, comparing dual-degree applicants to MD-only applicants. Subsequently, multivariable logistic regression was used, incorporating dual-degree status as an interaction term to evaluate whether the effects of these modifiers varied by degree path.

For specialty outcomes, the proportions of 23 medical specialties were analyzed for dual-degree groups in reference to MD-only. Specialties coded as preliminary or transitional were not included in specialty related analyses. Smaller specialties, such as medical genetics, vascular surgery, and thoracic surgery, had limited sample sizes and were excluded. A linear regression was used to evaluate the correlation between the specialty-specific ORs divided from TexasSTAR and those from the AAMC. For the AAMC data, ORs were calculated by comparing active MD-PhD residents to all residents without a PhD using Fisher’s pairwise exact tests.

Statistics were conducted using base R (version 4.2.2), car (v3.1-3), rstatix (v0.7.2), stats (v4.2.2), and MASS (v7.3-58.2) packages. Model assumptions for generalized linear mixed models were evaluated using the DHARMa package (v0.4.7). Data were formatted into tables using the finalfit (v1.0.8), gtsummary (v2.0.4), and flextable (v0.9.7) packages. The data were filtered and visualized using the tidyverse collection of packages (v2.0.0), ggeffects (v2.0.0), ggforestplot (v0.1.0), ggpubr (v0.6.0), and patchwork (v1.3.0).

### Study approval.

This study was reviewed and exempted by the IRB at the University of North Carolina.

### Data availability.

Raw data used in this study can be requested through the Texas STAR by emailing texasSTAR@utsouthwestern.edu or by contacting the deans of a participating US-accredited medical school: https://www.utsouthwestern.edu/education/medical-school/about-the-school/student-affairs/texas-star.html AAMC Report on Residents Table B4 can be downloaded from: https://www.aamc.org/data-reports/students-residents/data/report-residents/2024/table-b4-md-phd-residents-gme-specialty Doximity residency program rank lists can be viewed using their Residency Navigator: https://www.doximity.com/residency/ All code used to generate statistics and figures can be found in the publicly available GitHub repository: https://github.com/physicianscientists/Texas-STAR (CommitID: b051ffedecc2badc4f416fd2ea28f4b337748886). Data associated with the analyses are available in the Supporting Data Values file.

## Author contributions

All authors were involved in conceptualization, writing, and editing the original draft. DCB contributed to methodology, software, formal analyses, investigation, and visualization. DDR contributed to visualization. CSJ contributed to data curation. CYT contributed to supervision, methodology, investigation, data curation, visualization, and project administration. TD and EMW contributed to writing and editing the original draft.

## Funding support

- Funding for this study was provided by the American Physician Scientists Association.

## Supplementary Material

Supplemental data

Supplemental tables 1-2

Supporting data values

## Figures and Tables

**Table 1:**
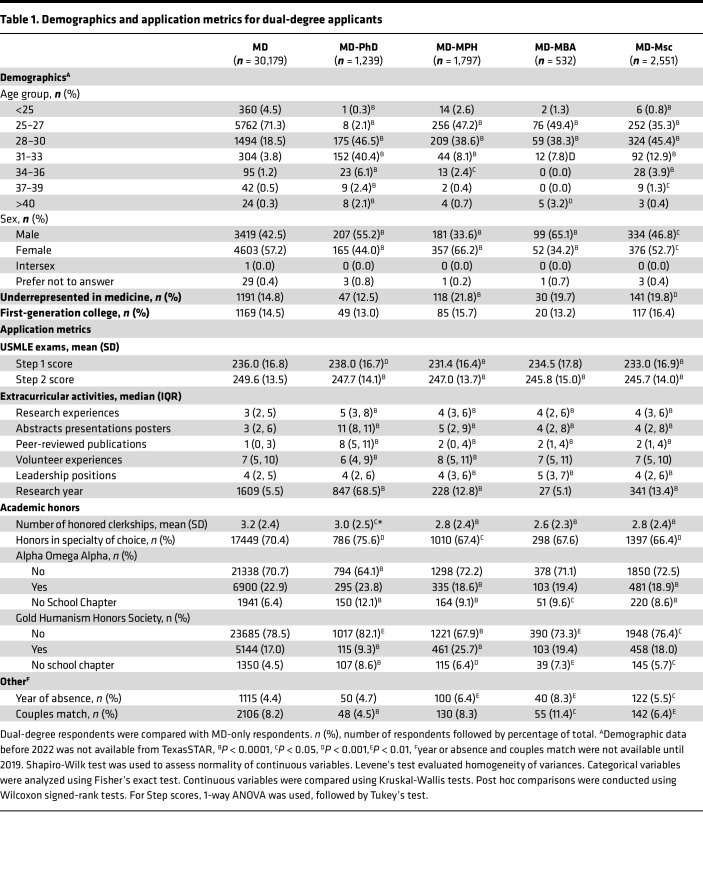
Demographics and application metrics for dual-degree applicants

**Table 2:**
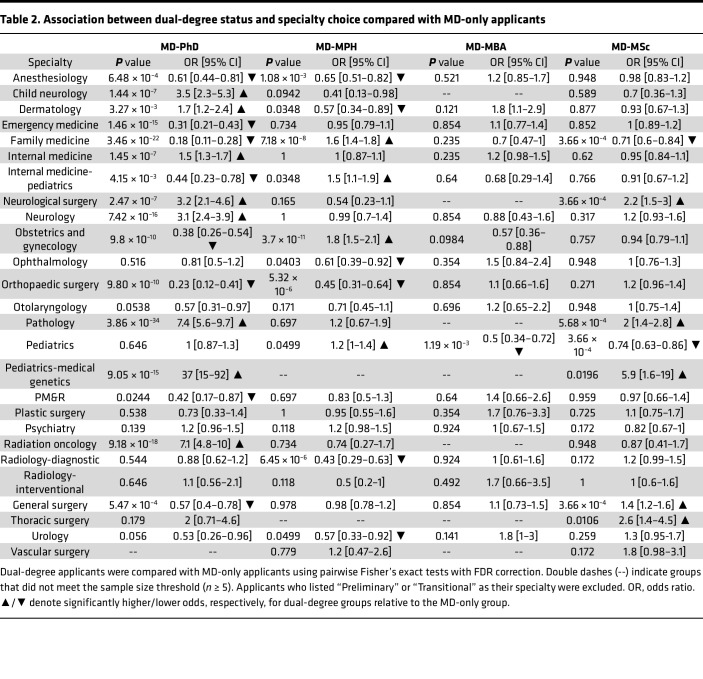
Association between dual-degree status and specialty choice compared with MD-only applicants

## Version 1 02/19/2026

In-Press Preview

## Version 2 04/08/2026

Electronic publication

## References

[1] Drum B (2023). Values-based resident selection in an internal medicine-pediatrics residency program. J Gen Intern Med.

[2] Hammoud MM (2021). The 2020-2021 residency application cycle: lessons learned and lingering problems. JAMA.

[3] Lipman JM (2023). A systematic review of metrics utilized in the selection and prediction of future performance of residents in the United States. J Grad Med Educ.

[4] Patel MS (2010). Match rates into higher-income, controllable lifestyle specialties for students from highly ranked, research-based medical schools compared with other applicants. J Grad Med Educ.

[5] Collins RA (2024). Characteristics associated with successful residency match in general surgery. Ann Surg Open.

[6] Saguil A (2012). The association between specialty match and third-year clerkship performance. Mil Med.

[7] Rinard JR, Mahabir RC (2010). Successfully matching into surgical specialties: an analysis of national resident matching program data. J Grad Med Educ.

[8] Benjamin WJ (2024). Evaluating the impact of the novel geographic preferences section on interview rate and residency match outcomes. J Gen Intern Med.

[9] Belkowitz J (2023). Early career outcomes of a large four-year MD/ MPH program: results of a cross sectional survey of three cohorts of graduates. PLoS One.

[10] Brass LF, Akabas MH (2019). The national MD-PhD program outcomes study: Relationships between medical specialty, training duration, research effort, and career paths. JCI Insight.

[11] Andriole DA (2008). Characteristics and career intentions of the emerging MD/PhD workforce. JAMA.

[12] Reilly JM (2021). Dual MD-MPH degree students in the United States: moving the medical workforce toward population health. Public Health Rep.

[13] Patel MS (2014). The role of MD and MBA training in the professional development of a physician: a survey of 30 years of graduates from the Wharton Health Care Management Program. Acad Med.

[14] Agarwal G (2024). Beyond the M.D.: Transdisciplinary approaches of high-volume dual degree M.D./Masters programs at U.S. allopathic medical schools. BMC Med Educ.

[15] Jeffe DB (2014). The emerging physician-scientist workforce: demographic, experiential, and attitudinal predictors of MD-PhD program enrollment. Acad Med.

[16] Laditi F (2023). Characterization of the landscape of joint MD/MBA programs in the US, 2002 to 2022. JAMA Netw Open.

[17] Russell AW (2008). Double degrees: double the trouble or twice the return?. High Educ.

[18] https://www.utsouthwestern.edu/education/medical-school/about-the-school/student-affairs/texas-star.html.

[19] Brass LF (2023). Physician-scientists in anesthesiology: the all too empty pipeline. Anesth Analg.

[20] Brass LF (2010). Are MD-PhD programs meeting their goals? An analysis of career choices made by graduates of 24 MD-PhD programs. Acad Med.

[21] von Kaeppler EP (2020). MD-PhD graduates remain underrepresented in orthopaedic surgery: national MD-PhD program outcome survey update. J Orthop Res.

[22] Harding CV (2017). History and outcomes of 50 years of physician-scientist training in medical scientist training programs. Acad Med.

[23] Vinagre R (2020). Red flags, geography, exam scores, and other factors used by program directors in determining which applicants are offered an interview for anesthesiology residency. Cureus.

[24] Gauer JL, Jackson JB (2017). The association of USMLE Step 1 and Step 2 CK scores with residency match specialty and location. Med Educ Online.

[25] Garber AM (2019). Use of filters for residency application review: results from the internal medicine in-training examination program director survey. J Grad Med Educ.

[26] Wissel BD (2022). MD-PhD students are underrepresented in the gold humanism honor society. Acad Med.

[27] https://www.doximity.com/residency.

[28] Romanoski NL (2025). The relative influence of program signaling, geographic preferences, and in-state status in determining odds of interview invitation in residency selection. J Grad Med Educ.

[29] https://www.aamc.org/data-reports/students-residents/report/facts.

[30] Tang CY (2025). Training physician-scientists, a view from inside. Nat Med.

[31] Christensen BR (2020). A comparison of match outcomes between traditional medical degree and dual-degree applicants. PLoS One.

[32] Mather RV (2024). Dispelling the myth: comparable duration and impact of research training for MD-PhD and PhD graduates. JCI Insight.

[33] Martinez-Strengel A (2022). Trends in U.S. MD-PhD program matriculant diversity by sex and race/ethnicity. Acad Med.

[34] Williams DKA (2024). Sociodemographic factors and research experience impact MD-PhD program acceptance. JCI Insight.

[35] Nguyen M (2024). Socioeconomic diversity in admissions to MD-PhD programs, 2014–2019. JAMA Netw Open.

[36] Cavanagh A (2023). Diversity in MD-PhD programs and factors affecting admission and completion among minoritized groups: a scoping review. Acad Med.

[37] Krupat E (2017). Medical students who pursue a joint MD/MBA degree: who are they and where are they heading?. Eval Health Prof.

[38] Andriole DA (2016). Characteristics and career intentions of MD-MPH program graduates: a national cohort study. Public Health Rep.

[39] Weng J (2025). 2024 Physician-Scientist Trainee Diversity Summit conference proceedings. J Clin Transl Sci.

[40] Christophers B (2021). First-generation physician-scientists are under-represented and need better support. Nat Med.

[41] Roghmann M-C (2025). An analysis of curricular structures in MD-PhD programs in the United States. Acad Med.

[42] Ozair A (2023). The US residency selection process after the United States medical licensing examination step 1 pass/fail change: overview for applicants and educators. JMIR Med Educ.

[43] Nasser JS (2023). Matching into competitive surgical residencies: predictors of success. Med Educ Online.

[44] https://www.nrmp.org/wp-content/uploads/2024/09/Program-Director-2024-Program-Signaling-Report-Final-09242024.pdf.

[45] https://www.nrmp.org/match-data/2025/05/results-and-data-2025-main-residency-match/.

[46] Smith BB (2018). Impact of Doximity Residency Navigator on Graduate Medical Education Recruitment. Mayo Clin Proc Innov Qual Outcomes.

[47] https://www.aamc.org/data-reports/data/eras-statistics-data.

[48] https://assets.doxcdn.com/image/upload/pdfs/residency-navigator-survey-methodology.pdf.

