# Supplementary material for: Dual-degree pathways in the residency match: a comparative analysis of application behaviors and outcomes
Source: JCI Insight. 2026 Feb 19;11(7):e198778. doi: 10.1172/jci.insight.198778 (PMC13134727; doi:10.1172/jci.insight.198778)
Supplement: Supplemental data [file jciinsight-11-198778-s025.pdf]

SUPPLEMENTAL FIGURES

Supplemental Figure 1

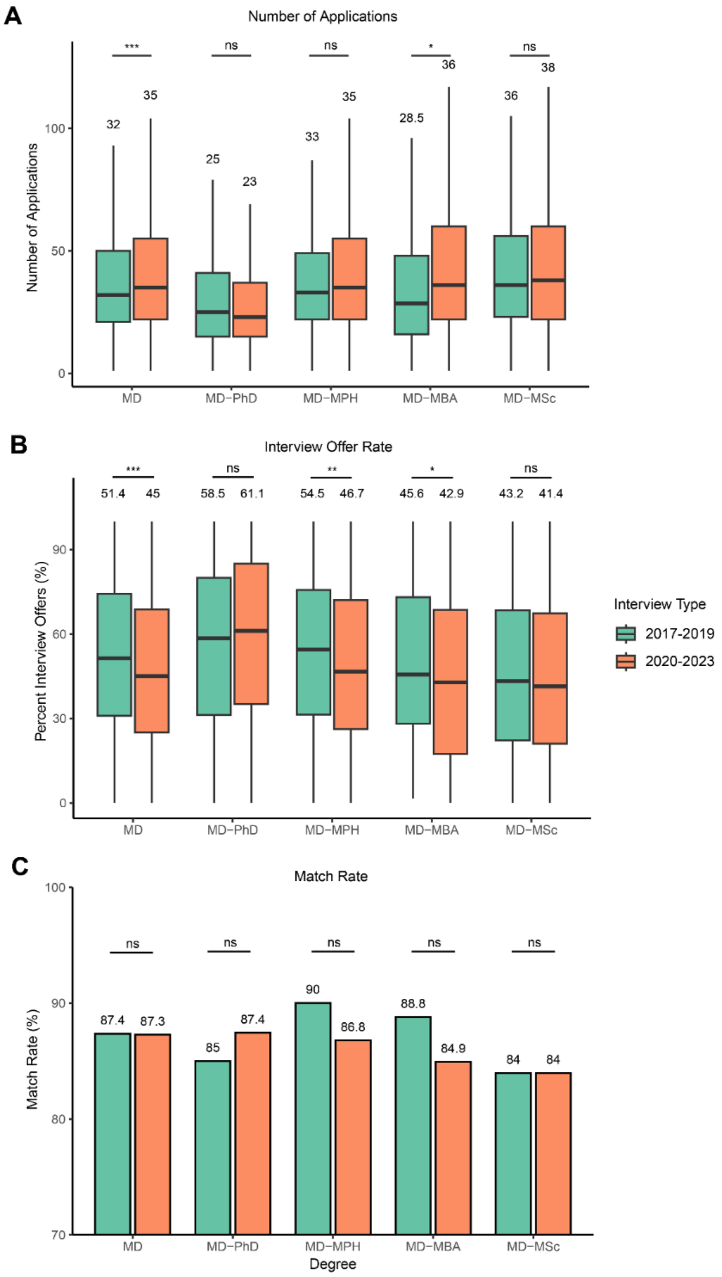

Supplemental Figure 1. Application, interview, and match trends before and after the COVID-19 pandemic. Comparison of the A) total number of residency applications submitted, B) interview offer rate, and C) match rate for each degree type, stratified by in-person interviews (green, 2017-2019) and shift to virtual interviews (orange, 2020-2023). Boxplots display the median and interquartile range (IQR), with whiskers extending to 1.5xIQR. Comparisons were evaluated using Wilcoxon signed rank tests for total number of residency applications submitted and interview offer rate, while Fisher's exact tests were used for match rate. *p<0.05, **p<0.01, ****p<0.001; ns, not significant.

Supplemental Figure 2

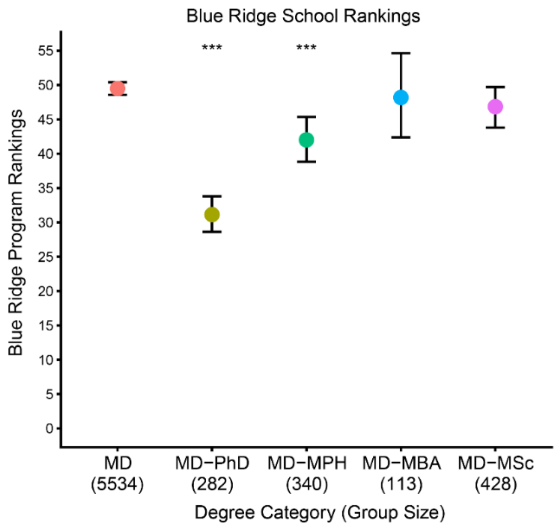

Supplemental Figure 2. Specialty-specific rankings of matched applicants. Mean residency program rankings, as defined by the Blue Ridge Institute for Medical Research, stratified by degree group. Points represent group means. Error bars indicate 95% confidence intervals. Statistical significance was assessed using pairwise Wilcoxon rank-sum tests comparing each dual-degree group with MD-only applicants. Lower Blueridge program ranking values reflect higher-ranked programs.

Supplemental Figure 3

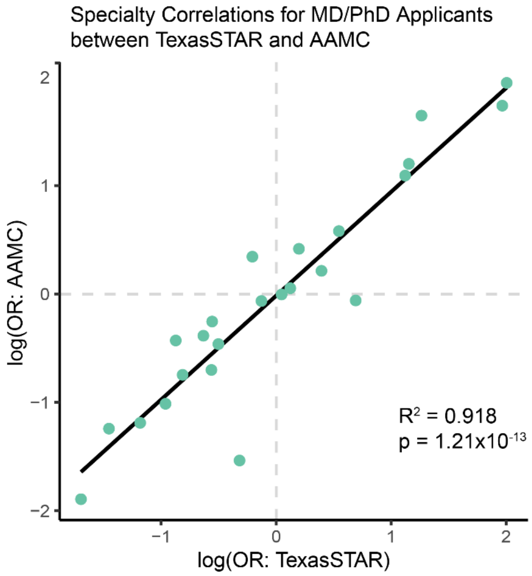

Supplemental Figure 3: Overall Concordance in Specialty Preference between TexasSTAR and AAMC for MD-PhD Students. Each point represents a medical specialty. The black diagonal line denotes the line of identity (y = x), indicating perfect alignment. Odds ratios and p-values are reported in Supplemental Table 2.
